# Comparative Efficacy of Esmolol, Labetalol, and Lignocaine in Attenuating Hemodynamic Responses to Laryngoscopy and Intubation: A Randomized Double-Blind Study

**DOI:** 10.7759/cureus.110768

**Published:** 2026-06-13

**Authors:** Rojalin Sahoo, Suryasnata Sahoo, Ananta Narayan Patra, Swatee Shatarupa Shubhadarshini

**Affiliations:** 1 Anesthesiology, Dharanidhar Medical College and Hospital, Odisha, IND; 2 Anesthesiology and Critical Care, Dharanidhar Medical College and Hospital, Odisha, IND; 3 Anesthesiology and Critical Care, Srirama Chandra Bhanja Medical College and Hospital (SCBMCH), Cuttack, IND

**Keywords:** endotracheal intubation, esmolol, hemodynamic response, labetalol, laryngoscopy, lignocaine, randomized controlled trial

## Abstract

Background

Laryngoscopy and endotracheal intubation are associated with significant sympathetic stimulation resulting in tachycardia and hypertension. These hemodynamic changes may be harmful, particularly in patients with limited cardiovascular reserve. Various pharmacological agents have been used to attenuate this response, but an ideal agent remains debatable.

Aim

To compare the efficacy of intravenous esmolol, labetalol, and lignocaine in attenuating peri-intubation hemodynamic responses, assessed using heart rate, systolic blood pressure, diastolic blood pressure, mean arterial pressure, and rate pressure product during laryngoscopy and endotracheal intubation

Materials and methods

Patients were randomly allocated into three equal groups (n = 25 each). Group ES received intravenous esmolol 1 mg/kg, Group LB received intravenous labetalol 0.25 mg/kg, and Group LG received intravenous lignocaine 1.5 mg/kg, administered two minutes prior to laryngoscopy and endotracheal intubation. Heart rate, systolic blood pressure, diastolic blood pressure, mean arterial pressure, and rate pressure product were recorded at baseline, after drug administration, at intubation, and at one, three, five, seven, and 10 minutes following intubation. Data were analyzed using repeated-measures ANOVA, one-way ANOVA, chi-square test, and Tukey post hoc analysis, with p < 0.05 considered statistically significant.

Results

Baseline demographic parameters and hemodynamic variables were comparable among the three groups. All three drugs attenuated the hemodynamic response to laryngoscopy and intubation to varying extents. Labetalol demonstrated the most effective attenuation of heart rate, systolic blood pressure, mean arterial pressure, and rate pressure product at intubation and throughout the post-intubation period (p < 0.001). Esmolol showed better attenuation compared to lignocaine but was less effective than labetalol. Lignocaine was the least effective agent. No significant adverse effects were observed in any group.

Conclusion

Intravenous labetalol is more effective than esmolol and lignocaine in attenuating the hemodynamic responses to laryngoscopy and endotracheal intubation, with better overall cardiovascular stability and safety.

## Introduction

Among the most physiologically stimulating events encountered during the perioperative period, direct laryngoscopy and orotracheal intubation consistently provoke a robust sympathoadrenal response. The mechanical stimulation of supraglottic structures and the upper trachea triggers a surge in circulating catecholamines, producing clinically measurable elevations in cardiac frequency, systolic and diastolic arterial pressures, and mean arterial pressure (MAP) [1]. In otherwise healthy surgical candidates, this cardiovascular perturbation is transitory and self-limiting. Nonetheless, in individuals burdened with pre-existing cardiac dysfunction, hypertensive disease, intracranial pathology, or cerebrovascular insufficiency, the consequences of unmitigated hemodynamic surges may be severe [2].

The clinical repercussions of exaggerated pressor responses during airway instrumentation have been thoroughly documented and encompass myocardial ischemia, life-threatening arrhythmias, acute cardiac decompensation, intracranial hemorrhage, and pathologically elevated intracranial pressure [3]. Consequently, the pharmacological modulation of these responses continues to occupy a central position in contemporary anesthetic practice. A diverse array of strategies has been explored, ranging from augmentation of anesthetic depth to the administration of opioid analgesics, vasodilating agents, calcium antagonists, and adrenergic blocking drugs [4].

Among pharmacological interventions, beta-adrenergic antagonists and sodium channel-blocking anesthetics have garnered particular interest owing to their established safety profiles and demonstrated efficacy. Esmolol, a highly cardioselective beta-1 blocker with an ultrashort half-life, has been recognized as a potent attenuator of peri-intubation tachycardia and a reducer of myocardial oxygen demand during stressful airway maneuvers [5]. Its rapid onset and swift offset confer distinct advantages in the dynamic intraoperative context. Labetalol, acting through concurrent blockade of both alpha-1 and beta-adrenergic receptors, integrates rate control with peripheral vascular relaxation, thereby producing a dual attenuation of both heart rate (HR) and systemic vascular resistance [6]. Its relatively prolonged pharmacodynamic duration may, however, constrain dose titration flexibility in certain operative settings.

Lignocaine (lidocaine), a membrane-stabilizing agent with established utility as a local anesthetic and class IB antiarrhythmic, has been evaluated for its capacity to blunt airway reflexes and sympathetic activation when delivered via the intravenous route [7]. Its primary mechanism involves blockade of fast sodium channels, thereby interrupting neural transmission and dampening catecholamine secretion. However, the extent to which intravenous lignocaine attenuates the pressor component of the intubation response has yielded discordant results across independent investigations [8].

Despite an abundance of studies evaluating each of these agents individually, head-to-head comparisons conducted under equivalent anesthetic conditions remain sparse. Therefore the aim of this study is to compare the efficacy of intravenous esmolol, labetalol, and lignocaine in attenuating peri-intubation hemodynamic responses, assessed using HR, systolic blood pressure (SBP), diastolic blood pressure (DBP), MAP, and rate pressure product (RPP) during laryngoscopy and endotracheal intubation.

## Materials and methods

Study setting

Following formal approval by the Institutional Ethics Committee, this prospective randomized double-blind trial was conducted within the Department of Anaesthesiology at S.C.B. Medical College and Hospital, Cuttack, over a 12-month period spanning November 2019 to November 2020.

Study population

The study cohort comprised 75 adult patients of both sexes, ranging in age from 20 to 60 years, with physical status categorized as Grade I or II according to the American Society of Anesthesiologists (ASA) classification, who were scheduled to undergo elective operative procedures necessitating general anesthesia with endotracheal intubation. A formal sample size calculation was not performed prior to study initiation because of limited institutional data regarding expected intergroup differences. A convenience sample of 75 patients (25 per group), comparable to previously published studies evaluating attenuation of intubation responses, was therefore included

Eligibility criteria

Eligible participants were adults aged 20-60 years with a body mass index not exceeding 30 kg/m2, classified as ASA physical status I or II, and capable of providing voluntary written informed consent. Subjects were excluded if they carried an ASA classification of III or above, were pregnant or lactating, or had a documented history of bronchial asthma, significant cardiovascular morbidity, or morbid obesity. Further exclusion criteria encompassed current use of antihypertensive pharmacotherapy, predicted difficult airway anatomy, laryngoscopy or intubation duration extending beyond 20 seconds, and known hypersensitivity to any of the investigational drugs.

Pre-anaesthetic assessment

A comprehensive pre-anesthetic evaluation was performed for every participant on the day preceding the scheduled procedure. This assessment encompassed clinical examination of the cardiovascular and respiratory systems, nutritional and general health status, Mallampati airway grading, and a thorough medication and allergy history. Laboratory investigations included complete blood count, hemoglobin estimation, bleeding and clotting times, ABO blood typing, urinalysis, 12-lead electrocardiography, plain chest radiography, fasting and two-hour postprandial plasma glucose, serum urea and creatinine, and electrolyte panel comprising sodium and potassium. A preoperative fasting period of eight hours was enforced. Oral premedication the night before surgery included alprazolam 0.25 mg and ranitidine 150 mg in tablet form.

Group allocation and randomization

The 75 enrolled participants were allocated at random into three equal arms of 25 subjects each through a sealed-envelope randomization system: Group LG receiving intravenous lignocaine (1.5 mg/kg), Group ES receiving intravenous esmolol (1 mg/kg), and Group LB receiving intravenous labetalol (0.25 mg/kg). Each study drug was prepared in an identical syringe volume of 10 mL by a pharmacist uninvolved in data collection, preserving blinding for both the treating anesthesiologist and the patient. The randomization sequence was generated using a computer-generated random number table by an independent investigator not involved in patient recruitment or data collection. Allocation concealment was ensured using sequentially numbered opaque sealed envelopes, which were opened immediately before preparation of the study drug

Anesthetic protocol

Peripheral intravenous access was secured with an 18-gauge cannula and Ringer's lactate infusion commenced. Continuous monitoring included cardiac rate, non-invasive blood pressure, pulse oximetry, and five-lead electrocardiography. Baseline recordings of SBP, DBP, MAP, and HR were documented. All subjects received intravenous glycopyrrolate (0.05 mg/kg), midazolam (0.05 mg/kg), and nalbuphine (0.2 mg/kg) 10 minutes before administration of the study drug. Anesthesia was induced following three minutes of preoxygenation with 100% oxygen, using thiopentone sodium (5 mg/kg) and vecuronium bromide (0.1 mg/kg). Orotracheal intubation was performed with a cuffed tube of appropriate size. Anesthetic maintenance employed a nitrous oxide-oxygen mixture supplemented with isoflurane and intermittent vecuronium. To minimize operator-related variability, all laryngoscopies and endotracheal intubations were performed by experienced anesthesiologists with comparable expertise, and intubation duration was restricted to less than 20 seconds

Hemodynamic surveillance

Hemodynamic parameters including HR, SBP, DBP, MAP, and RPP were documented at the following standardized time points: at baseline, immediately prior to intubation (after drug administration), at the moment of intubation, and at one, three, five, seven, and 10 minutes following intubation. The primary outcome of the study was attenuation of the peri-intubation hemodynamic response, assessed by changes in HR and MAP following laryngoscopy and endotracheal intubation. Secondary outcomes included SBP, DBP, RPP, and the incidence of adverse events.

Definition of adverse events

Hypotension was defined as SBP falling below 90 mmHg or declining by more than 30% from the baseline value. Hypertension was identified when SBP exceeded 150 mmHg or surpassed the baseline by more than 30%. Tachycardia was designated when HR exceeded the baseline value by more than 25%. A heart rate below 60 beats per minute was classified as bradycardia. Any departure from normal sinus rhythm constituted dysrhythmia. Post-operative observation in the post-anesthesia care unit (PACU) continued for two hours, with surveillance for nausea, emesis, hypotension, bradycardia, excessive sedation, and other untoward events. No clinically significant hypotension, bradycardia, dysrhythmia, excessive sedation, or other adverse events requiring intervention were observed in any study group during intraoperative or PACU monitoring.

Statistical methods

All statistical analyses were performed using SPSS version 22.0 (IBM Corp., Armonk, NY, USA) and R version 3.2 (R Foundation for Statistical Computing, Vienna, Austria). Continuous variables are presented as mean ± standard deviation, while categorical variables are expressed as frequencies and percentages. Baseline demographic and clinical characteristics were compared among groups using one-way analysis of variance (ANOVA) for continuous variables and the chi-square test or Fisher's exact test for categorical variables, as appropriate.

Repeated hemodynamic measurements (HR, SBP, DBP, MAP, and RPP) obtained at multiple time points were analyzed using repeated-measures ANOVA, with time as the within-subject factor and treatment group as the between-subject factor. Main effects of group and time, as well as group × time interaction effects, were evaluated. Assumptions of normality were assessed using the Shapiro-Wilk test, and sphericity was evaluated using Mauchly's test. When the sphericity assumption was violated, Greenhouse-Geisser correction was applied.

Where significant overall differences were identified, post hoc pairwise comparisons were performed using Tukey's honestly significant difference test with adjustment for multiple comparisons. A two-tailed p-value < 0.05 was considered statistically significant, while p < 0.001 was considered highly significant.

## Results

All 75 randomly assigned participants completed the study protocol. Three groups of 25 individuals were formed: the lignocaine group (LG), the esmolol group (ES), and the labetalol group (LB), with complete data available for analysis from every enrolled subject.

Baseline demographic profile

The mean ages for the LG, ES, and LB groups were 39.08 ± 10.25, 38.72 ± 11.09, and 35.20 ± 10.04 years, respectively. One-way ANOVA confirmed no statistically significant difference in age distribution (F = 0.13, p = 0.876). A slight male predominance was observed across the entire cohort (56%), and sex distribution was equivalent across the three arms (chi-square = 0.32, p = 0.850). Mean body weights were 53.64 ± 7.73 kg, 56.04 ± 7.28 kg, and 56.72 ± 8.52 kg for the LG, ES, and LB groups, respectively, with no intergroup difference reaching significance (F = 0.30, p = 0.741). These findings confirm demographic homogeneity across the study arms (Table 1).

**Table 1:** Age Distribution of Patients in the Three Groups

Age (years)	Lignocaine n (%)	Esmolol n (%)	Labetalol n (%)	Total n (%)
21–30	8 (32%)	7 (28%)	9 (36%)	24 (32%)
31–40	8 (32%)	8 (32%)	9 (36%)	25 (33.3%)
41–50	4 (16%)	5 (20%)	3 (12%)	12 (16%)
51–60	5 (20%)	5 (20%)	4 (16%)	14 (18.7%)
Total	25	25	25	75
Mean ± SD	39.08 ± 10.25	38.72 ± 11.09	35.20 ± 10.04	37.67 ± 10.49

Heart rate response

Pre-drug baseline HRs were statistically comparable across all three groups (p > 0.05). Laryngoscopy and intubation provoked a marked rise in HR in every arm; however, the magnitude of this elevation differed significantly. The LG group exhibited the steepest increase, reaching a peak of 110.40 ± 5.74 bpm at the time of intubation, compared with 106.16 ± 6.40 bpm in the ES group and 97.00 ± 10.70 bpm in the LB group. The LB group maintained a significantly attenuated HR response relative to both ES and LG from the moment of intubation through all subsequent measurement intervals (p < 0.001). Repeated measures ANOVA demonstrated a highly significant intergroup difference in HR across time (F = 18.42, p < 0.001), with Tukey post hoc analysis confirming labetalol's superiority at each post-intubation time point (Table 2).

**Table 2:** Pulse Rate (beats/min) ANOVA + Tukey test: heart rate significantly lower in the labetalol group at all post-intubation intervals (p < 0.001)

Time	Lignocaine	Esmolol	Labetalol
Baseline	82.40 ± 8.81	82.20 ± 8.97	80.08 ± 8.74
Drug given	80.64 ± 8.85	80.24 ± 8.91	77.28 ± 8.60
Intubation	110.40 ± 5.74	106.16 ± 6.40	97.00 ± 10.70
1 min	107.76 ± 5.83	103.76 ± 6.50	92.92 ± 10.84
3 min	103.52 ± 5.99	99.28 ± 6.73	87.60 ± 7.34
5 min	98.32 ± 6.08	94.04 ± 7.17	83.52 ± 6.10
7 min	93.12 ± 6.35	88.32 ± 7.87	80.00 ± 5.00
10 min	87.20 ± 6.97	84.24 ± 8.21	78.44 ± 4.08

Systolic blood pressure

Pre-intubation SBP was equivalent among the three groups (p > 0.05). Following airway instrumentation, all groups demonstrated significant SBP elevation, with the sharpest increase recorded in the LG group (152.60 ± 6.27 mmHg). The ES and LB groups recorded intubation SBP values of 144.40 ± 3.67 mmHg and 140.04 ± 6.58 mmHg, respectively. Both esmolol and labetalol conferred statistically significant SBP attenuation relative to lignocaine throughout the intubation and immediate post-intubation periods (p < 0.05). At the majority of post-intubation assessments, labetalol demonstrated superior SBP control over esmolol. One-way ANOVA confirmed significant intergroup variation (F = 22.15, p < 0.001), with Tukey analysis establishing labetalol's advantage over both lignocaine (p < 0.001) and esmolol (p < 0.05) (Table 3).

**Table 3:** Systolic Blood Pressure (mmHg) Labetalol showed superior systolic blood pressure control (p < 0.05)

Time	Lignocaine	Esmolol	Labetalol
Baseline	117.88 ± 6.92	117.76 ± 7.13	118.56 ± 4.60
Drug given	115.96 ± 6.96	115.80 ± 6.95	116.52 ± 4.67
Intubation	152.60 ± 6.27	144.40 ± 3.67	140.04 ± 6.58
1 min	150.44 ± 6.31	141.00 ± 4.00	138.40 ± 6.31
3 min	145.20 ± 6.47	135.28 ± 4.33	130.36 ± 7.09
5 min	137.80 ± 6.06	129.48 ± 5.39	127.36 ± 6.93
7 min	130.00 ± 6.65	124.08 ± 6.22	121.88 ± 6.42
10 min	123.60 ± 6.62	119.84 ± 6.85	114.52 ± 5.72

Diastolic blood pressure

Baseline DBP values did not differ meaningfully across the three groups (p > 0.05). Intubation produced a marked DBP elevation in all arms, with the LG group showing the largest absolute rise. Both esmolol and labetalol significantly curtailed this response relative to lignocaine at the time of intubation and one minute thereafter (p < 0.05). Beyond the first post-intubation minute, DBP differences between esmolol and labetalol did not achieve statistical significance (p > 0.05), suggesting broadly equivalent diastolic pressure control following resolution of the initial hemodynamic surge. ANOVA at intubation yielded F = 16.08, p < 0.001 (Table 4).

**Table 4:** Diastolic Blood Pressure (mmHg)

Time	Lignocaine	Esmolol	Labetalol
Baseline	76.28 ± 6.49	76.40 ± 6.59	76.56 ± 7.54
Drug given	74.40 ± 6.35	74.68 ± 6.44	74.44 ± 7.34
Intubation	125.12 ± 5.37	117.60 ± 6.65	113.12 ± 7.50
1 min	120.60 ± 6.04	114.88 ± 6.03	110.08 ± 7.96
3–10 min	Comparable in all groups		

Mean arterial pressure

Baseline MAP was homogeneous across groups. Airway instrumentation resulted in pronounced MAP elevation in all subjects, with the greatest rise recorded in the LG group (134.20 ± 4.36 mmHg at intubation). ES and LB groups recorded values of 126.60 ± 4.39 mmHg and 124.68 ± 6.87 mmHg, respectively. Both active comparators significantly outperformed lignocaine in attenuating MAP at intubation and during the early post-intubation window (p < 0.05). At selected time intervals, labetalol demonstrated marginally superior MAP regulation relative to esmolol. All reported SBP, DBP, and MAP values were rechecked against the original study dataset, and no transcription errors were identified. ANOVA revealed significant intergroup variation (F = 19.76, p < 0.001), with Tukey analysis confirming labetalol's advantage over both lignocaine (p < 0.001) and esmolol (p < 0.05) (Table 5).

**Table 5:** Mean Arterial Pressure (mmHg)

Time	Lignocaine	Esmolol	Labetalol
Baseline	90.04 ± 6.60	92.60 ± 9.90	90.56 ± 6.50
Intubation	134.20 ± 4.36	126.60 ± 4.39	124.68 ± 6.87
1 min	130.52 ± 4.76	123.60 ± 3.84	122.84 ± 7.19
10 min	95.40 ± 4.16	94.40 ± 5.57	91.88 ± 3.72

Rate pressure product

Baseline RPP was statistically equivalent across all three groups (p > 0.05). Following intubation, RPP rose significantly in every arm, with the LG group recording the highest peak (22030.96 ± 1263.21 mmHg/min), followed by the ES group (15332.16 ± 1044.09 mmHg/min) and the LB group (13635.60 ± 2076.02 mmHg/min). Both esmolol and labetalol significantly reduced RPP relative to lignocaine (p < 0.001), with labetalol demonstrating consistently superior myocardial protection across most post-intubation intervals. ANOVA confirmed highly significant intergroup variation (F = 28.64, p < 0.001), and Tukey analysis identified labetalol as significantly superior to both esmolol (p < 0.01) and lignocaine (p < 0.001) (Table 6).

**Table 6:** Rate Pressure Product (RPP)

Time	Lignocaine	Esmolol	Labetalol
Baseline	9776 ± 1217	9676 ± 1174	9495 ± 1110
Intubation	22030 ± 1263	15332 ± 1044	13635 ± 2076
1 min	20907 ± 1348	14663 ± 1055	12897 ± 1834
5 min	15062 ± 1100	12175 ± 1053	10659 ± 1187
10 min	10583 ± 994	10069 ± 1113	8969 ± 597

## Discussion

The act of direct laryngoscopy and passage of an endotracheal tube constitutes one of the most potent sympathetic stimuli encountered in routine anesthetic practice. The resultant catecholamine surge manifests clinically as tachycardia and acute hypertension, increasing the risk of adverse perioperative cardiac events. Pharmacological attenuation of this response assumes particular urgency in patients with pre-existing cardiovascular compromise. The current prospective, randomized, double-blind trial set out to determine which of three commonly administered agents - esmolol, labetalol, or lignocaine - most reliably and safely suppresses these hemodynamic perturbations.

Comparable baseline characteristics across the three treatment groups, encompassing age, sex distribution, body weight, and resting hemodynamic parameters, affirm the internal validity of the study and lend confidence that post-intubation differences arose from the pharmacological interventions themselves rather than from patient-level confounders.

The labetalol group exhibited the most pronounced suppression of HR elevation following laryngoscopy and endotracheal intubation, maintaining substantially lower rates throughout all post-intubation time points compared with both comparators. This outcome reflects the compound pharmacodynamic profile of labetalol: simultaneous blockade of peripheral alpha-1 adrenoceptors and cardiac beta-adrenoceptors reduces sympathetic outflow to the vasculature while directly constraining chronotropic drive. These findings align with prior reports by Singh et al. [6] and Sharma et al. [9], who similarly identified labetalol as superior to beta-selective agents in controlling intubation-related tachycardia.

Esmolol achieved meaningful HR attenuation relative to lignocaine, though falling short of labetalol's efficacy. Its targeted beta-1 selectivity predominantly influences cardiac chronotropy without substantially altering peripheral vascular tone, which may account for its incomplete pressor blunting at standard dosages. This interpretation is consistent with earlier observations by Miller et al. [10] and Korpinen et al. [11], who noted that esmolol's anti-tachycardic effect may require higher doses to extend meaningfully to the blood pressure dimension.

Lignocaine proved least effective in moderating HR among the three agents examined. The transient duration of its sodium-channel blockade and unpredictable modulation of catecholamine secretion likely underlie its suboptimal performance. Kindler et al. [12] drew comparable conclusions, reporting that intravenous lignocaine offered only marginal protection against intubation-induced tachycardia.

Regarding SBP, lignocaine was again the least effective, while both labetalol and esmolol produced statistically significant pressor attenuation. The peripheral vasodilatory effect of labetalol's alpha-1 blockade provides a mechanistic explanation for its superior SBP control, a conclusion reinforced by other studies which documented enhanced blood pressure management with labetalol during airway manipulation [6,13].

For DBP, both esmolol and labetalol outperformed lignocaine during the critical peri-intubation window. The convergence of DBP values between esmolol and labetalol beyond the first post-intubation minute indicates that these two agents achieve comparable diastolic control once the initial adrenergic surge subsides.

MAP trends mirrored those observed for SBP, with labetalol providing the closest approximation to baseline MAP values throughout the measurement period. Its capacity to reduce MAP without precipitating clinically meaningful hypotension illustrates a favorable therapeutic index for perioperative use. 

The RPP, employed here as a non-invasive surrogate for myocardial oxygen demand, was most effectively reduced by labetalol. This finding carries direct clinical implications for patients predisposed to myocardial ischemia, in whom even brief episodes of elevated myocardial oxygen consumption may precipitate adverse events. Ansari et al. [13] similarly highlighted the importance of minimizing RPP elevation during airway instrumentation in high-risk populations.

The tolerability profile of all three agents was acceptable, with no clinically significant hypotension, bradycardia, or dysrhythmias necessitating intervention in any group. Labetalol's combination of cardiovascular efficacy and a reassuring safety profile at the doses employed supports its preferential selection as a pretreatment agent before laryngoscopy and intubation.

Limitations

Several methodological constraints merit acknowledgment. The cohort size was relatively modest, and enrollment was restricted to ASA physical status I and II patients, limiting extrapolation to higher-risk clinical populations. As a single-center investigation, site-specific practices may have influenced outcomes in ways not generalizable to other institutional settings. Fixed drug dosing precluded exploration of dose-response relationships, and hemodynamic surveillance was confined to the 10 minutes immediately following intubation, leaving longer-term cardiovascular effects uncharacterized. The exclusive use of non-invasive blood pressure monitoring may have introduced measurement imprecision when compared with direct intra-arterial recordings. Additionally, a formal sample size power calculation was not performed prior to study initiation, which may limit precision in estimating treatment effects. Potential operator-related variability may also have influenced peri-intubation hemodynamic responses despite standardized intubation techniques. Furthermore, adverse events were infrequent, limiting detailed comparative safety analysis between groups.

## Conclusions

Among the three pharmacological agents evaluated in this study, intravenous labetalol demonstrated the most effective attenuation of the hemodynamic stress response associated with direct laryngoscopy and endotracheal intubation in ASA I-II patients undergoing elective surgery. Compared with esmolol and lignocaine, labetalol provided superior control of HR, SBP, DBP, MAP, and RPP without clinically significant adverse effects. Within the study population and dosing regimen evaluated, labetalol was the most effective agent among the three drugs studied. However, larger multicenter studies involving broader patient populations are required to confirm these findings and establish their generalizability.
